# Novel methodology to assess sputum smear microscopy quality in private laboratories

**DOI:** 10.1186/1471-2334-12-331

**Published:** 2012-11-29

**Authors:** Andrew J Codlin, Mona Javaid, Fahad Qazi, Mishal S Khan

**Affiliations:** 1 Interactive Research and Development (IRD), Suite 508, Ibrahim Trade Tower, Main Shahrah-e-Faisal, Karachi, 75350, Pakistan; 2 Indus Hospital, Korangi Crossing, Karachi, 75190, Pakistan; 3 Current address: Research Alliance for Advocacy and Development (RAAD), Karachi, Pakistan; 4 Current address: London School of Hygiene and Tropical Medicine, Keppel Street, London, WC1E 7HT, UK

**Keywords:** Tuberculosis, Diagnosis, Sputum smear microscopy, Private sector, EQA, Pakistan

## Abstract

**Background:**

In South Asia, it is estimated that 80% of patients choose to attend a private facility for their healthcare needs. Although patients generally believe that the private-sector provides high quality services, private diagnostic laboratories are largely unregulated and little is known about the accuracy of results provided. This study assesses the accuracy of sputum smear microscopy for pulmonary tuberculosis diagnosis in private laboratories operating in Karachi, Pakistan. A novel evaluation methodology was designed in which patient-actors submitted sputum specimens spiked with cultured *Mycobacterium tuberculosis* (Mtb) for testing such that laboratories were not aware that they were being assessed.

**Methods:**

Smear-negative sputum specimens from Indus Hospital TB Program patients were collected and combined with an attenuated, cultured Mtb strain to create Mtb-spiked samples; for negative standards, no Mtb was added to the smear-negative sputum specimens. Seven of the largest private laboratories across Karachi were chosen for evaluation and were sent six Mtb-spiked and one Mtb-negative sputum specimens. Patient-actors pretending to be laboratory customers submitted these specimens to each laboratory for testing over a three day period.

**Results:**

Only three laboratories accurately classified all the Mtb-spiked specimens which were submitted. A further three misclassified all the Mtb-spiked specimens as smear-negative, thus providing the ‘patients’ with false negative results.

**Conclusions:**

TB sputum smear microscopy services are highly variable across private laboratories and are often of extremely poor quality. Engagement, capacity building and rigorous monitoring of standards at private laboratories are of vital importance for the control of TB. Our findings, while specific for TB diagnostic tests, could be symptomatic of other tests performed in private laboratories and warrant further investigation.

## Background

Despite improvements in the functioning of government-run tuberculosis (TB) control programs over the past two decades, case detection still falls short of the Millennium Development Goal target of diagnosing 70% of new TB cases. It is estimated that 35% of all incident TB cases, which amounts to over 3 million infected individuals, were not detected by TB control programs in 2010 [1]. It is now widely recognized that strengthening public-sector TB diagnostic and treatment services alone is not enough to reduce transmission of TB. Engaging with private health facilities, and understanding the strengths and weaknesses of the services they provide to TB patients, is crucial to improving case detection and treatment.

South Asia in particular has experienced a huge expansion in the private healthcare sector over the past two decades, resulting in the region having the highest rate of private-sector healthcare utilization globally. An estimated 80% of all patients in the region attend private facilities for their healthcare needs, including TB diagnostic and treatment services [2,3]. Private healthcare facilities are utilized by all groups of society, including the poorest, owing to better privacy, accessible locations, more convenient opening hours and perceived higher-quality care compared with government-run facilities.

Pakistan, which has the fifth-highest TB burden globally, is a prime example of a country with an active private health sector [1]. Reports indicate that for each patient visit to a public-sector healthcare facility in Pakistan, there are more than three visits to a private practitioner [2]. Private allopathic health facilities are found mainly in cities, and comprise small clinics run by trained or untrained general practitioners, large hospitals, diagnostic laboratories and pharmacies. All of these private facilities are visited by TB suspects and numerous public private mix (PPM) programs have successfully increased TB case detection and notification through their engagement [4-6]. However, the scalability and replicability of these programs have yet to be demonstrated. Currently, less than 30 private-sector facilities based in Karachi, a city of over 20 million, are reporting TB cases to the Provincial TB Program, Sindh.

Despite the size of the private healthcare sector in South Asia, little is known about the quality of services offered by private establishments. Reports indicate that general practitioners often prescribe inappropriate drug regimens to treat TB [7,8] and that TB diagnostic tests at private laboratories are substandard [9]. In Pakistan, as in most low- and middle-income countries, sputum smear-microscopy is the most common test used to confirm TB infection due to its limited cost and equipment requirements. However, the test is heavily dependent on the microscopy technician and involves several steps, all of which must be performed correctly, to obtain reliable results [9]. To monitor and improve the quality of smear-microscopy at diagnostic centres, the National Tuberculosis Control Programme (NTP) of Pakistan runs an External Quality Assurance (EQA) program for all diagnostic centres that collaborate with them on case notification. The EQA program includes site inspections of laboratory facilities, panel testing, and collection and blinded rereads of saved slides to check that initial slide reading and reporting was accurate. Currently, most private sector TB diagnostic facilities in Pakistan fall outside the scope of these EQA efforts, and many do not agree to participate in quality assurance procedures.

The objective of this study was to assess the accuracy of sputum smear microcopy services in private laboratories in Karachi, Pakistan. As all currently accepted EQA methods require the knowledge and consent of the facility being evaluated, a novel methodology was designed in which patient-actors submitted sputum specimens spiked with *Mycobacterium tuberculosis* (Mtb) to private laboratories, allowing for laboratory reported results to be compared against the known smear-microscopy results.

## Methods

### Specimen collection and preparation

The sputum specimens used for this study were collected from TB suspects at the Indus Hospital TB Control Program clinic following standard hospital procedures. Though it was recently established and is entirely funded by grants and donations, the Indus Hospital TB Control Program is the highest volume TB treatment center in Karachi. The hospital currently serves as a reference lab for the programmatic management of drug-resistant tuberculosis for the province of Sindh.

Sputum specimens were first processed using standard Ziehl-Neelsen (ZN) staining and sputum smear microscopy techniques to determine their smear status. To make Mtb-spiked sputum specimens, smear-negative sputum specimens were inoculated with cultured Mtb as follows. Fresh cultures of the attenuated Mtb strain H37Rv (ATCC #27294) were grown on Löwenstein-Jensen (LJ) slants and then transferred into sterile 7H9 broth. Glass beads (1-2mm) were added and the suspension was vortexed to make a homogenous bacterial suspension, with an absorbance greater than 1 McFarland. The suspension was allowed to stand undisturbed for 20 minutes so clumped Mtb could fall out of solution. The supernatant was then transferred to a new test tube. The suspension in the second test tube was allowed to stand undisturbed for a further 15 minutes, before the supernatant was transferred to a third test tube. The suspension turbidity was then adjusted to 0.5 McFarland by adding additional 7H9 broth. 100μL of the 0.5 McFarland Mtb suspension was added to 5mL of smear-negative sputum and the suspension was vortexed to create a spiked sputum specimen with a 1+ designation. For Mtb negative standards, 7H9 broth containing no cultured Mtb was added to the sputum specimen.

In total, 56 Mtb-spiked sputum specimens were made: 6 smear-positive and 1 smear-negative specimens for each laboratory targeted in this evaluation. ZN staining and sputum smear microscopy was performed by the Indus Hospital laboratory manager on these specimens to confirm their classification as smear-positive or -negative before they were transported to any private laboratories. For safe transport, Mtb-spiked sputum specimens were loaded by the Indus Hospital laboratory into sterile, twist-cap containers and packed inside a sealed cooler box.

### Private lab identification and specimen submission

Seven high-volume private laboratories were chosen for inclusion in this evaluation. Four laboratories are part of an extensive network system where biological specimens are sent to a central branch for testing from satellite sites located throughout the city. The remaining three laboratories are standalone facilities. Together, these seven labs represent the majority of private laboratory services in Karachi.

Three patient-actors were recruited, after taking informed consent to participate in the study. They were instructed on biosafety measures to avoid accidental infection, including that they were not to open any of the twist-cap containers and to return to the hospital without touching any contaminated material should any of the containers leak during transport. A cell phone was provided to the patient-actors to monitor their progress and trouble shoot any issues that arose once they left the hospital. The patient-actors visited each laboratory and posed as customers, with assumed names, ages and general practitioner diagnostic test prescriptions. They paid for sputum smear microscopy tests in the same way as all other laboratory customers and submitted the spiked sputum specimens over a three day period, such that each lab under evaluation received 7 specimens. Additionally, 7 specimens were sent to the Indus Hospital laboratory for blinded testing. De-identified laboratory IDs were assigned to each private laboratory which reflect the relative cost of sputum smear microscopy among the evaluated laboratories, with 1 being the most and 7 the least expensive.

Test results from the private laboratories were picked up by the patient-actor the day after each specimen was submitted. Results were entered into an Access database and frequencies of correct classification were calculated using SAS 9.0 (Cary, North Carolina).

This study acquired ethical approval from the Office for Human Research Protections (OHRP) registered institutional review board of Indus Hospital (no. IRD_IRB_2011_9_003). All sputum specimens were strictly de-identified and were obtained from the Indus Hospital laboratory after routine test results had reported back to clinicians and patients and the specimens were flagged for disposal.

## Results

Table 1 indicates the frequency and percent of correct sputum smear classification at each laboratory. Only two of seven private laboratories correctly classified all the specimens which were submitted, with four laboratories correctly classifying less than half of submitted specimens. For several laboratories, only the Mtb negative specimens were correctly classified. In light of this finding, the percentage of correctly identified Mtb-spiked and Mtb-negative specimens (respectively representing the sensitivity and specificity of sputum smear microscopy at each laboratory) are shown. Three laboratories correctly classified all of the Mtb-spiked specimens, while three others incorrectly classified all the Mtb-spiked specimens as smear-negative. Laboratory 6 was the only facility to incorrectly classify an Mtb-negative specimen as smear-positive. Indus Hospital laboratory technicians did not know that they were under evaluation or that the specimens were laboratory-made, yet they correctly classified all submitted specimens.

**Table 1:** Frequency of correct specimen classification

**Laboratory ID**	**Laboratory type**	**Correct diagnosis**		
		**All Specimens, n (%)**	**Smear-Positive Specimens, n (%)**	**Smear-Negative Specimens, n (%)**
1	Network	3 (42.9)	2 (33.3)	1 (100)
2	Network	7 (100)	6 (100)	1 (100)
3	Standalone	1 (14.3)	0 (0)	1 (100)
4	Standalone	1 (14.3)	0 (0)	1 (100)
5	Network	1 (14.3)	0 (0)	1 (100)
6	Standalone	6 (85.7)	6 (100)	0 (0)
7	Network	7 (100)	6 (100)	1 (100)
Indus Hospital	N/A	7 (100)	6 (100)	1 (100)

## Discussion

To our knowledge, this is the first study to assess the quality of TB diagnostic services at private laboratories by sending Mtb-spiked sputum specimens for testing, with the facility under review being unaware of the evaluation. Our results show that sputum smear microscopy services in the seven largest private laboratories in Karachi are variable and often of extremely poor quality. Four out of the seven laboratories assessed provided incorrect results to the patient-actors, stating that their sputum did not contain TB bacteria, when in fact, it was an Mtb-spiked specimen. Further, three of the seven laboratories provided false negative results for all six Mtb-spiked specimens that were submitted for testing. The finding that several laboratories are consistently failing to identify bacteria in sputum specimens known to contain Mtb is surprising and raises important concerns.

It is likely that a large number of infectious TB cases are going undiagnosed at these laboratories because patients and their physicians are receiving results falsely stating that submitted sputum specimens are free of Mtb. Although physicians likely ordered a sputum smear microscopy test based on clinical and/or radiological suggestions of TB, upon receiving false negative results, they may delay initiating TB treatment or unduly rule out TB as the patient’s diagnosis. As TB patients with bacteria in their sputum are both more infectious and more likely to have a poor prognosis compared with smear-negative TB patients, the receipt of inaccurate test results from a diagnostic laboratory is likely to have an adverse impact on the patient and his/her contacts.

Additionally, this study highlights the need for rigorous assessment and regulation of TB diagnostic services, and potentially other diagnostic services, offered in the private sector. Test results from private laboratories are generally perceived by both patients and physicians to be more reliable than those from over-burdened, public facilities; often a (correct) smear-positive diagnosis from a government centre will be disregarded if a negative result from a private laboratory is received. Because such a large number of patients seek healthcare in the private sector in South Asia, it is important that governments take steps to ensure at least a minimal standard of service provision. For private laboratories in particular, it is very difficult for a patient to distinguish between laboratories that provide poor quality versus high quality diagnostic services, particularly as it appears that higher cost is not associated with improved accuracy of results. Without any regulation and enforcement of standards, laboratories may have more incentive to cut costs and compromise on service quality, potentially allowing them to either competitively lower their prices or increase their profit margins.

It must be acknowledged that this novel method of assessing AFB smear microscopy quality has a different purpose and role than traditional EQA methods. Standard EQA methods allow the assessor to identify the stage in the sputum smear microscopy process at which an error is being made. However, in order to conduct the EQA, the laboratory must agree to be assessed and the laboratory technicians may be aware of the assessment. Both of these features of EQA pose major disadvantages when assessing private laboratories, as in Pakistan and many other developing country contexts, these facilities are not bound by law to cooperate with government EQA programs. Often the laboratories that require the most training and regulation are those that decline to participate in EQA, knowing the extra work they may have to take on as a result of participation. Furthermore, even when laboratories agree to participate in EQA programs, the results of their EQA may not be representative of the quality of their work under normal conditions.

Using this novel methodology, it is not possible to identify the reason for the inaccurate sputum smear microscopy results. The laboratories we assessed may have provided false negative results due to any of the following reasons: incorrect fixing of sputum specimens to slides, incorrect staining procedures, poor microscopy techniques, insufficient time spent on reading slides or even failure to process the specimen at all. However, a major benefit of this methodology is that it allows the assessment of any laboratory that accepts specimens from walk-in patients.

This study and methodology are not without potential limitations. The number of laboratories included and specimens sent to them was relatively small, limiting the ability of these results to be generalized. However, the largest, most well-known private laboratories in Karachi, which together with their satellite specimen collection sites make up the majority of private laboratory services in the city, were evaluated by this initiative. Further, this assessment was conducted immediately before a PPM active-case finding project aimed at improving private laboratory diagnostic services started activities; a more comprehensive, baseline study with additional specimens and varied amounts of bacilli was impossible because the PPM project conducted site inspections and smear microscopy training at several of the sites under review. Despite these limitations, this methodology was able to identify poor quality smear microscopy services at private laboratories that are unwilling to participate in traditional EQA programs.

Mtb has the tendency to clump together in sputum and it is possible that some labs would select a section of the Mtb-spiked sputum specimen to read which does not contain bacteria. To minimize this from occurring, spiked specimens were created and thoroughly homogenized the morning they were submitted to private laboratories. The fact that specimens were sent to the laboratories over a three day period and that reported results were either very accurate or very inaccurate, suggests that laboratory procedures, rather than random clumping of specimens, were responsible for the differences found.

## Conclusions

Despite the small sample size of this study, its findings are both interesting and troubling. The size of the private healthcare sector in South Asia makes it an essential partner for TB care, yet many of its institutions offer substandard care. Continued engagement, capacity building and rigorous monitoring of standards at private laboratories are of vital importance for the control of TB. Though our findings are specific for TB diagnostic tests, they could be symptomatic of other tests performed in private laboratories and warrant further investigation.

## Competing interests

The authors declare that they have no competing interests.

## Authors’ contributions

AJC participated in the study’s design, coordination, data analysis and helped draft the manuscript. MJ helped design and implement all laboratory related aspects of this study. FQ participated in the study design and the drafting of the manuscript. MK conceived of the study and participated in the study’s design and manuscript preparation. All authors read and approved the final manuscript.

## Pre-publication history

The pre-publication history for this paper can be accessed here:

http://www.biomedcentral.com/1471-2334/12/331/prepub
